# From Pain to Paralysis: Shingles Crosses the Line

**DOI:** 10.7759/cureus.87168

**Published:** 2025-07-02

**Authors:** Mazhar Alam, Omar Shafey

**Affiliations:** 1 Internal Medicine/Acute Medicine, Blackpool Victoria Hospital, Blackpool, GBR; 2 Medicine, Bedfordshire Hospital NHS Foundation Trust, Luton, GBR

**Keywords:** herpes zoster, monoparesis, rash, shingles, zoster-associated limb paresis

## Abstract

We report a rare case of monoparesis due to herpes zoster infection in an 83-year-old female. Weakness in the woman’s upper limb started soon after the appearance of a vesiculo-papular rash over the left shoulder area extending down into the arm. Investigations were essentially normal apart from herpes zoster DNA being detected in PCR testing of a swab taken from the skin lesions. Imaging studies ruled out other possible causes of weakness like stroke and brachial plexus injury. The patient was commenced on antiviral treatment, which led to gradual improvement in the weakness. This is one of the lesser-known complications of herpes zoster, which can have a significant impact on quality of life. Also called zoster-associated limb paresis (ZALP), it can affect 0.5-5% of herpes zoster cases.

## Introduction

Herpes zoster, commonly known as shingles, is a reactivation of the varicella-zoster virus (VZV), which typically causes chickenpox during primary infection. Following this initial episode, the virus stays dormant in the dorsal root ganglia. It can reactivate later in life, particularly in immunocompromised or elderly individuals [1-3].

While shingles most often presents with painful vesicular rash localized to a dermatome, which is an area of skin receiving sensory innervation from the dorsal root of a single spinal nerve, complications can extend beyond the cutaneous manifestations. Among the less common but clinically significant complications is segmental zoster paresis, a rare condition characterized by motor weakness in the myotome (a group of muscles innervated by a single spinal nerve root) corresponding to the affected dermatome [4].

Segmental zoster paresis is estimated to occur in 3-5% of patients with herpes zoster [5], and its clinical presentation can be easily mistaken for other neurological conditions such as a stroke or brachial plexopathy. When shingles affects the cervical dermatomes, particularly C5 to C7, motor involvement may manifest as acute flaccid paralysis of the upper limb. This condition may develop days to weeks after the onset of the characteristic rash, and in some cases, motor deficits may precede or occur without cutaneous symptoms, further complicating the diagnosis.

This case report presents an unusual instance of left upper limb paralysis following herpes zoster involving the left cervical dermatomes in an otherwise immunocompetent adult.

## Case presentation

An 83-year-old woman presented to the hospital with weakness affecting her left upper limb for the past four days. She gave an interesting history of having an accidental fall 10 days prior, sustaining a mild head injury and some straining to her left arm. To relieve the pain, she had applied ibuprofen gel to her left shoulder area but stopped doing so, as she noticed a rash of tiny spots erupting over the area. The patient initially thought it was a reaction to the ibuprofen gel, yet the rash kept extending into her arm, and a few days later, she started noticing weakness in the limb. At that point, she consulted a doctor.

**Figure 1:**
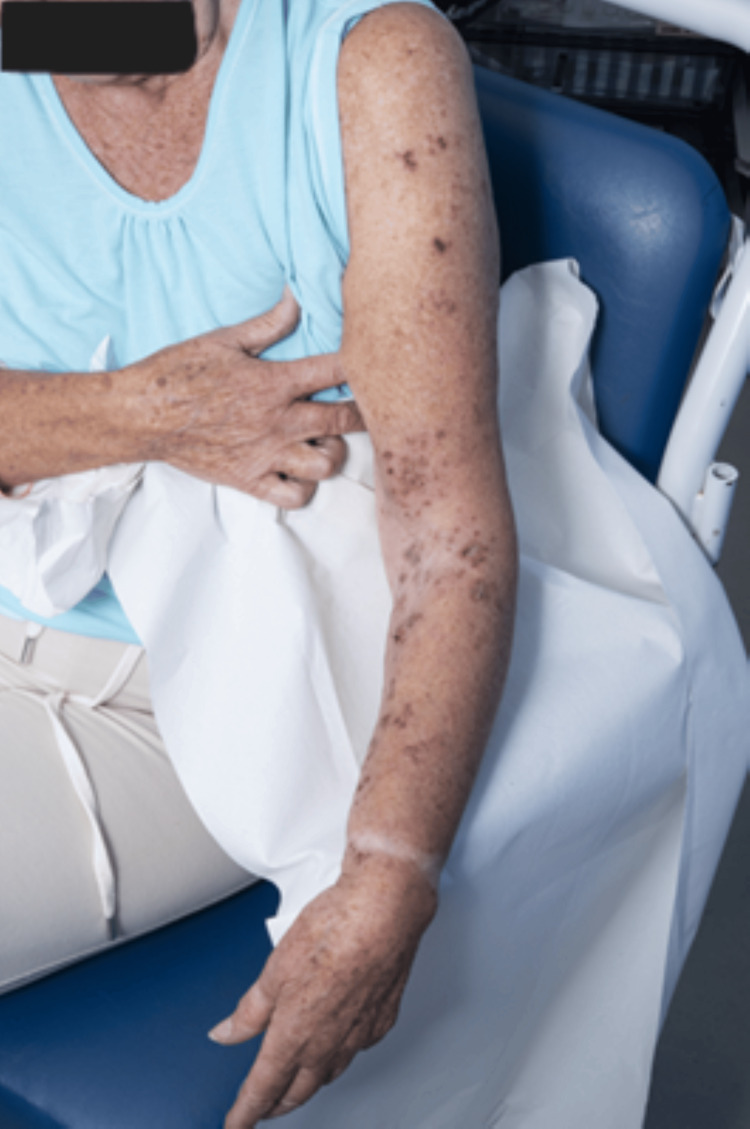
Rash over her left arm healing on discharge from the hospital

On examination, the patient had a slightly painful vesiculo-papular rash over the left shoulder area extending down into the arm (Figure 1). Neurological examination demonstrated markedly reduced power in the left upper limb with shoulder abduction 1/5, elbow extension and flexion 2/5, and wrist extension 4/5 with reduced grip in the left hand. The muscle bulk was preserved, yet tone was reduced, and the deep tendon reflexes in the affected limb were absent. She had a subtle sensory loss in the C5, C8, and T1 distributions on the left as well. 

The initial impression was that of traumatic brachial plexus injury due to the fall, and the rash was due to NSAIDs. Vasculitis was also considered in the differential or a possible cerebrovascular accident. Management plan included routine blood tests; X-rays of the left shoulder and elbow; nerve conduction studies; MRI of the head, neck, and brachial plexus; and vasculitis screening. These investigations did not reveal much apart from a subtle increased signal intensity of the left-sided brachial plexus cords compared with the right side, with no signs of neuritis or root injury.

Dermatology advice was sought, who suggested herpes zoster as the cause of the rash affecting the C5 and C6 dermatomes, advising oral antiviral treatment. Subsequently, herpes zoster DNA was detected in PCR testing of a swab taken from a skin lesion.

Considering the presentation and in light of the various investigations, a provisional diagnosis of zoster-associated limb paresis (ZALP) was made, and neurology input was requested. The neurologist confirmed herpes zoster brachial plexopathy as the diagnosis and advised an extended course of antivirals (valaciclovir 1000 mg three times a day for an extended period of two weeks) and physiotherapy for the left upper limb weakness.

We followed up with the patient after discharge. She is generally doing well after more than a year since she suffered from the condition, yet still has residual weakness in her left upper limb, predominantly around her shoulder, preventing her from raising her arm above her shoulder height.

## Discussion

The incidence of herpes zoster is approximately 4-4.5 per 1000 person-years [2,3]. It can lead to numerous complications, including postherpetic neuralgia, encephalitis, aseptic meningitis, and ZALP. ZALP is a rare yet significant complication of herpes zoster that can affect 0.5-5% of cases of herpes zoster [3,6,7]. The pathogenesis of ZALP is unclear; however, it may be due to the virus spreading into the anterior horn of the spinal cord from the dorsal root ganglia, where it had been lying dormant [6]. The motor weakness typically begins after two to three weeks of the rash eruption. This is a characteristic of a lower motor neuron lesion and can cause muscle atrophy. Most cases of ZALP occur on the face (50%) but can rarely affect the extremities [6]. If the sacral region is affected, it can lead to bowel and bladder dysfunction. Muscle weakness gradually resolves in most cases over a period of time.

Early antiviral therapy with valaciclovir has been shown to reduce the severity and duration of herpes zoster symptoms and may prevent complications such as post-herpetic neuralgia and paresis. In this case, the patient was started on valaciclovir promptly, which is standard for the treatment of herpes zoster.

ZALP may be particularly debilitating in the elderly and usually requires prolonged rehabilitation. Strengthening exercises and motor retraining can help restore function and improve the quality of life in these patients.

## Conclusions

This case highlights the importance of early recognition and management of herpes zoster and its potential complications, particularly in elderly patients. Post-herpetic paresis can significantly impact functional independence and requires a multidisciplinary approach, including antiviral therapy, physical rehabilitation, and regular follow-up to monitor progress. Early neurological specialty input and further electrophysiological investigations may help such cases in assessing the extent of nerve involvement and guiding further treatment strategies.
